# Enzyme-assisted extraction, characterization, and *in vitro* antioxidant activity of polysaccharides from *Potentilla anserina L*.

**DOI:** 10.3389/fnut.2023.1216572

**Published:** 2023-07-17

**Authors:** Penghui Guo, Hong Chen, Jinpu Ma, Yuxuan Zhang, Hongfu Chen, Ti Wei, Dandan Gao, Jiansheng Li

**Affiliations:** ^1^College of Life Sciences and Engineering, Northwest Minzu University, Lanzhou, China; ^2^Taizishan Ecosystem Observatory of Carbon Neutralization, Northwest Minzu University, Lanzhou, China; ^3^Nephropathy Department, Gansu Provincial Hospital of Traditional Chinese Medicine, Lanzhou, China

**Keywords:** *Potentilla anserina L*., polysaccharides, enzyme, extract, structural analysis, antioxidant activity

## Abstract

**Introduction:**

Potentilla anserina (*Potentilla anserina L*.), also known as ginseng fruit, is a plant that can be used as both medicine and food. *Potentilla anserina L*. has high medical value in Chinese medicine, such as strengthening the spleen and stomach, replenishing qi and blood, and astringing hemostasis.

**Methods:**

In this study, polysaccharides of *Potentilla anserina L*. were extracted from the root using an enzyme-assisted extraction method. According to the principle of Box–Behnken design, response surface methodology was designed to optimize the extraction conditions. Fourier transform infrared spectroscopy and scanning electron microscopy were used to investigate the structure and appearance of *Potentilla anserina L*. polysaccharides. The monosaccharide composition of *Potentilla anserina L*. polysaccharides was determined using high-performance liquid chromatography. The antioxidant activities were also studied.

**Results:**

Under the optimal extraction conditions (the ratio of solid to liquid, 1:15; ratio of cellulase to pectinase, 1:2; extraction pH, 8.0; enzyme reaction temperature, 60°C), the extraction yield of *Potentilla anserina L*. polysaccharides was 19.80 ± 0.01%, equal to the model prediction value 19.84%. The data of Fourier transform infrared spectrum, scanning electron microscopy, and high-performance liquid chromatography showed that the *Potentilla anserina L*. polysaccharide was a kind of α-pyran polysaccharide, mainly consisting of galactose, glucose, rhamnose, and arabinose. The antioxidant results showed that *Potentilla anserina L*. polysaccharides had a strong hydroxyl radical scavenging ability (IC_50_ = 0.367 mg/mL), superoxide anion scavenging ability (IC_50_ = 45.017 mg/mL), and a certain degree of total reducing ability.

**Discussion:**

Enzyme-assisted extraction is an efficient method to extract *Potentilla anserina L*. polysaccharides. The *Potentilla anserina L*. polysaccharides could have potential use in functional foods as a natural antioxidant.

## 1. Introduction

*Potentilla anserina L*. is known as ginseng fruit, also called “poke ma” or “Zhuo Lao Sha Zeng” in Tibetan. *Potentilla anserina L*. belongs to the Rosaceae family and is a typical stoloniferous and rosulate clonal plant (1). This plant is mainly distributed in cold and high-altitude areas in China, such as Gansu, Qinghai, and Tibet (2). *Potentilla anserina L*. has been consumed as medicine and food for more than 1,500 years (3). Its tuberous roots contain polysaccharides, succinic acid, triterpenoids, polyphenols, flavanols, flavonoids, triterpenoids, and other active substances. Therefore, *Potentilla anserina L*. has many physiological effects including liver protection, strengthening the spleen and stomach, and anti-tumor activities (3). *Potentilla anserina L*. has become a kind of popular tonic food and is applied in herbal medicine.

Polysaccharides widely exist in living organisms. This substance is composed of the same or different monosaccharides with α-or β-glycosidic bonds (4). Plant polysaccharides, also known as plant aggregate polysaccharides, are generally recognized as a healthy material to enhance disease resistance and reduce medication. Plant polysaccharides have health effects including immune regulation, hypoglycemia, and liver protection (5–7). To date, plant polysaccharides have been a research focus. The international scientific community puts forward that the 21^st^ century is the century of polysaccharides (8). *Potentilla anserina L*. polysaccharides (PAPs) have a variety of physiological and pharmacological properties, such as enhancing immunity and improving antioxidant capacity (9, 10).

Hot water extraction, ultrasonic-assisted extraction, ultrasonic-assisted two-phase extraction, and other methods were generally adopted to extract PAPs. These methods have at least one of the limits of requiring high temperature, long extraction time, high cost, low efficiency, and generating high noise. The enzyme-assisted method can be performed under a relatively mild condition with a short extraction time (2, 11). Enzyme-assisted extraction also has the advantages of simple operation and no pollution.

In this study, enzyme (cellulase and pectinase)-assisted extraction method was used to extract PAPs from *Potentilla anserina L*. root. The optimal extraction conditions were obtained by the response surface methodology, and the structure of PAPs was determined using the Fourier transform infrared spectrum (FT-IR) and scanning electron microscopy (SEM), and the monosaccharides' composition of PAPs were analyzed using high-performance liquid chromatography (HPLC). Moreover, the antioxidant activities of PAPs were also investigated by *in vitro* antioxidant experiments, which would provide a reference for the development and utilization of PAPs as a high-quality medicinal resource and functional food material.

## 2. Materials and methods

### 2.1. Materials and reagents

*Potentilla anserine L*. roots were purchased from the local market of Lanzhou, Gansu Province, China. The roots were crushed by a BJ-400 small high-speed reducing machine (Yongkang Boou Instrument Co., Ltd., Zhejiang, China) and processed by 100 mesh sieving. The sieved powder (100 g) was defatted with 500 mL of hexane for 3 h at 20°C and stirred continuously. After removing hexane, the powder was dried at room temperature.

Cellulase (3 U/mg) and pectinase (40 U/mg) were purchased from Solarbio Biological Reagent Co., Ltd. (Beijing, China). Potassium ferricyanide, trifluoroacetic acid, monosaccharide standard products, and acetonitrile were purchased from Sigma-Aldrich Chemical Co., Ltd. (Louis, USA). Other reagents were all analytically pure and purchased from Sinopharm Chemical Reagent Co., Ltd. (Beijing, China).

### 2.2. Preparation of PAPs

A measure of 3 g of pretreated *Potentilla anserine L*. root powder was accurately weighed and added to distilled water with a certain material–liquid ratio (1:10, 1:15, 1:20, 1:25, and 1:30). The hybrid of cellulase and pectinase (1:3, 1:2, 1:1, 2:1, and 3:1) was added into the suspension, and the pH (5.0, 6.0, 7.0, 8.0, and 9.0) was adjusted to a certain value for 90 min at various temperatures (40, 50, 60, 70, and 80°C). Then, the mixture was incubated in a water bath of 90°C for 5 min to deactivate the enzyme, centrifuged at 4,500 r/min for 5 min using a Heraeus Multifuge X1R centrifuge (Thermo Co., America). Sevage reagent (chloroform: n-butanol, 5: 1, v/v) was used to remove protein. Then, four volumes of anhydrous ethanol were added into the protein-free solution and left overnight at 4°C, which was then centrifuged at 4,500 rpm for 15 min. The recovered precipitate was dried in the DHG-9030A high-temperature oven (Shanghai Grows Instruments Co., Ltd., China).

The PAPs' extraction yield was calculated using the following equation (1):


(1) $$\begin{array}{l} R=\frac{{\text{m}}_{1}}{m_{0}}\times 100 \end{array}$$

In the formula, R represents the extraction yield of PAPs (%); m_1_ and m_0_ represent the weight of extracted PAPs and the weight of *Potentilla anserine L*. root powder (g).

### 2.3. Optimization of the extraction conditions

We investigated the effects of cellulase and pectinase ratio, pH, extraction temperatures, and solid–liquid ratio on the extraction yield of PAPs. The single-factor experiment was used to optimize the parameters one by one, and the extraction yield of PAPs was used as the evaluation indicator. Each experiment was conducted three times. Then, the three factors, which significantly influenced the yield, were selected as variables. Taking the extraction yield of PAPs as the response value, a three-factor and three-level response surface analysis test was generated to optimize the extraction process of PAPs. The experimental design of three-factor and three-level response surface is shown in Table 1. The linear quadratic model was fitted by the experiment results according to equation (2):


(2) $$\begin{array}{l} Y=B_{0}+\overset{n=3}{\underset{i=1}{\sum }}B_{i}X_{i}+\overset{n=3}{\underset{i=1}{\sum }}B_{ii}X_{i}^{2}+\overset{n=3}{\underset{i=1}{\sum }}B_{ij}X_{i}X_{j} \end{array}$$

In the formula, Y represents the response variable (PAPs' extraction yield, %); B_0_, B_i_, B_ii_, and B_ij_ represent the regression coefficients of variables for the intercept, linear, quadratic, and interaction terms, respectively; X_i_ and X_j_ represent the independent variables (j≠i).

**Table 1:** Process parameters setting for *Potentilla anserina L*. polysaccharides extraction, according to the Box–Benkhen design.

**Factor**	**Level**		
	−**1**	**0**	**1**
X_1_-enzyme ratio	1:1	1:2	1:3
X_2_-extraction pH	7.0	8.0	9.0
X_3_-extraction temperature/°C	50	60	70

### 2.4. FT-IR spectrometry of PAPs

A measure of 50 mg KBr and 0.5 mg PAPs were mixed and ground for 10 min and sieved. The mixture was pressed for 5 min using a HYP-15 press machine (Tianjin Guangdong Science and Technology Co., Ltd., Tianjin, China). The sample pieces were put into the sample chamber of a FT-IR-650 spectrometer (Tianjin Port East Technology Co., Ltd., China), and the spectral conditions of 4,000–400 cm^−1^ were set and scanned for 20 min to analyze the chemical structure of polysaccharides (12).

### 2.5. Scanning electron microscope experiment of PAPs

The PAPs sample was fixed on the sample table with electric tape, and the sample is plated with gold film by ion sputtering. The treated sample was placed into the sample chamber of a ZEISS-EVO18 tungsten wire scanning electron microscope (Carl Zeiss AG, Bruker Co., Germany) at conditions of 20 kV, and magnification was adjusted to observe the apparent morphology of PAPs (13).

### 2.6. Monosaccharide composition of PAPs

The monosaccharide composition of PAPs was determined using the reported HPLC method (14, 15) with a slight modification. In particular, a 10.00 mg PAPs' sample was dissolved in 5 mL of 2 mol/L of trifluoroacetic acid and treated at 100°C for 5 h. The pH was adjusted to 7.0 using 3 mol/L NaOH and then centrifuged (5,000 rpm, 10 min). A measure of 1 mL of the supernatant was taken, mixed with 0.2 mL of 0.5 mol/L of 1-phenyl-3-methyl-5-pyrazolone/methanol, and incubated at 70°C for 1 h to derivatize the sample. A measure of 0.2 mL of 0.3 mol/L of NaOH was added to neutralize the derivatization solution, and 1 mL of chloroform and 0.2 mL of HCl (0.3 mol/L) were added in the mixture, and then the solution was passed through a 0.22-μm filter membrane to obtain the solution. The mixed standard solution (galactose, rhamnose, fructose, arabinose, and glucose) was processed in the same way.

The analysis of monosaccharide components of PAPs was performed using the Agilent 1,260 HPLC chromatograph (Agilent, USA). The instrument was equipped with the diode array detector (DAD) and the Agilent ZORBAX Eclipse XDB-C18 column (4.6 × 250 mm, 5 μm). Acetonitrile and 20 mmol/L of phosphate buffer (pH 6.8) were selected as the mobile phase with a ratio of 19:81 (v/v, %), and the flow rate was 0.8 mL/min. The detection wavelength was 250 nm, the injection volume was 5 μL, and the column temperature was 28°C.

### 2.7. Antioxidant activity *in vitro*

#### 2.7.1. Determination of ·OH radical scavenging

According to the Fenton-type reaction of Wang et al., we determined the ·OH scavenging activity of PAPs (16) as follows: 1 mL of PAPs' solution (0.2, 0.4, 0.6, 0.8, and 1.0 mg/mL), 1 mL of 9 mmol/L of FeSO_4_ solution, 1 mL of 9 mmol/L of salicylic acid solution, and 1 mL of 8.8 mmol/L of H_2_O_2_ solution were added into a test tube and then mixed. The test tubes were incubated at 37°C for 30 min. The absorbance of all the samples was measured at 510 nm. Each sample was tested in triplicate, and the OH clearance rate of PAPs was calculated using the following formula (3).


(3) $$\begin{array}{l} E=\left(1-\frac{A_{1}-A_{2}}{A_{0}}\right)\times 100 \end{array}$$

In the formula, A_1_ represents the absorbance of the sample; A_2_ represents the absorbance value when distilled water was used instead of H_2_O_2_ solution, and other conditions remained unchanged; A_0_ represents the absorbance value of the blank group with distilled water instead of polysaccharide solution, and 0.2, 0.4, 0.6, 0.8, and 1.0 mg/mL of V_C_ solutions were used as positive controls.

#### 2.7.2. Determination of total reducing capacity

The reducing capacity was determined using the method of Hafsa et al. (17). In brief, 1 mL of PAPs' solution, 2.5 mL of sodium phosphate buffer (pH 6.6), and 2.5 mL of 1% potassium ferricyanide were mixed and kept at 50°C for 20 min. Thereafter, 2.5 mL of 10% trichloroacetic acid was quickly added and then centrifuged at 5,000 rpm for 10 min. A measure of 5 mL of the supernatant, 5 mL of distilled water, and 1 mL of 0.1% FeCl_3_ were mixed and reacted at room temperature, and then the absorbance of the reaction was determined at 700 nm. The same concentration of V_C_ solution was used as the control group. The strength of the reducing capacity was expressed as the magnitude of the absorbance value.

#### 2.7.3. Determination of ·O${}_{2}^{-}$ radical scavenging

Referring to the pyrogallol autoxidation method of Liu et al., the ·O${}_{2}^{-}$ scavenging activity of PAPs was measured as follows (18): 1 mL of PAPs' solution and 4.5 mL of 1 mol/L of Tris–HCl (pH 8.2) were mixed and placed at 50°C for 30 min. Then, 1 mL of 2 mmol/L of pyrogallic acid was added and kept at room temperature for 10 min. Finally, two drops of 8 mol/L of HCl were added to stop the reaction, and the absorbance value was measured at 320 nm (A_a_). A measure of 1 mL of distilled water was used to replace the PAPs' solution for the blank control, and the absorbance value of 320 nm was measured (A_b_). V_C_ was used as the positive control. The clearance activity was calculated as equation (4):


(4) $$\begin{array}{l} E=\frac{A_{a}-A_{b}}{A_{a}}\times 100 \end{array}$$

### 2.8. Statistics analysis

The experimental data were analyzed using the origin 2018 and SPSS 25.0 software (SPSS, Chicago, United States). The response surface experiment results were analyzed with Version 8.0.6 Design Expert software (Stat-Ease, Inc., Minneapolis, MN, USA). A one-way analysis of variance (ANOVA) was used to analyze the significance between groups, and all experiments or analyses were repeated three times.

## 3. Results and discussion

### 3.1. Effects of cellulase–pectinase ratio, pH, temperature, and solid–liquid ratio

**Figure 1:**
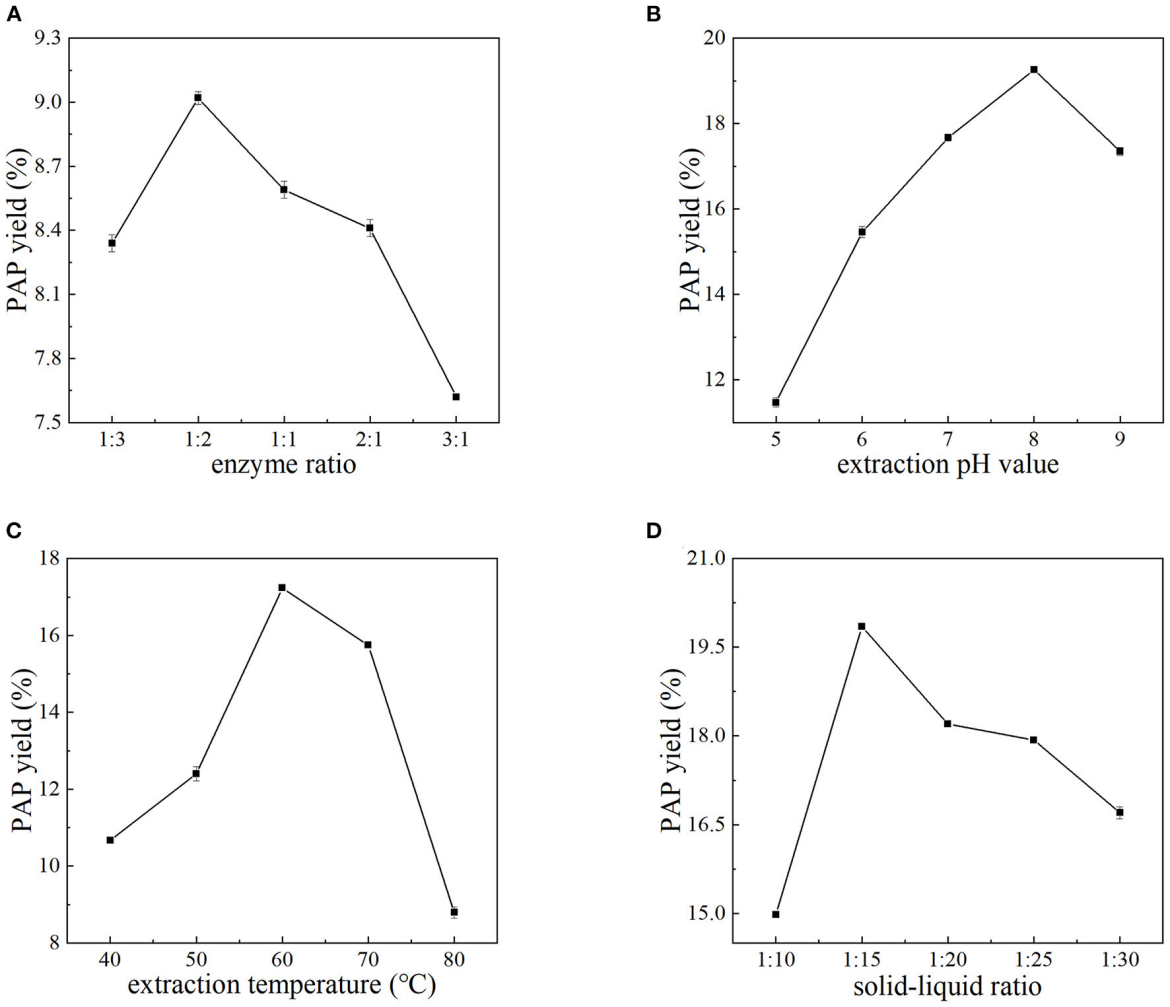
Effects of the ratio of cellulase to pectinase **(A)**, extraction pH **(B)**, extraction temperature **(C)**, and solid–liquid ratio **(D)** on the yield of PAPs.

In this section, the pH, extraction temperature, and solid–liquid ratio were fixed at 7.0, 60°C, and 1:15, respectively. The effects of the ratio of cellulase to pectinase on the yield of PAPs were investigated. The results are summarized in Figure 1A. As shown, by increasing the ratio of cellulase to pectinase from 1:3 to 3:1, the extraction rate of PAPs first increased and then decreased. In this investigation, the yield of PAPs was highest (9.02 ± 0.03%) at a ratio of 1:2, and then declined with further increasing the ratio. Cellulase can hydrolyze the cellulose of *Potentilla anserine* cell wall, reducing mass transfer resistance and accelerating the dissolution of PAPs (19). Pectinase can degrade the pectin of *Potentilla anserine* cell wall (20). The decrease in PAPs' extraction yield may be interpreted by the composition of *Potentilla anserine* cell wall. The cellulose content of the fern cell wall is higher than that of pectin, so an appropriately high ratio of cellulase to pectinase is needed. In this study, the maximum extraction yield of PAPs was obtained when the ratio of cellulase to pectinase was 1:2.

Figure 1B shows the relationship between the extraction yield of PAPs and pH value. The extraction yield of PAPs continued to increase with the pH increasing from 5.0 to 8.0. A further increase in pH decreased the yield instead. At pH 8.0, the extraction yield reached up to 19.26 ± 0.05%. Environmental pH is a pivotal factor modulating the enzyme activity, because the change of pH will affect the charge of the enzyme and the substrate, the degree of substrate dissociation, the active center of the enzyme, etc. (21, 22). When the pH was largely deviated from 8.0, the enzyme activity was restrained or the dissolubility of PAPs was decreased (22), leading to the decline of PAPs' extraction yield. The optimal pH value for the reaction system was 8.0.

It is obvious from Figure 1C that the extraction yield of PAPs showed a linear upward trend as the extraction temperature increased from 40 to 60°C. The PAPs' extraction yield reached the highest 17.24 ± 0.08% at 60°C. However, the extraction yield decreased with the temperature increasing from 60 to 80°C. An appropriate temperature can promote the diffusion of the enzyme molecules, thus increasing enzyme loading (23). An appropriate temperature promoted the dissolution of PAPs (24). A higher temperature may degenerate the enzyme, thus inhibiting the enzyme activity. In this study, 60°C seemed to be the proper temperature.

Figure 1D shows the relationship between the extraction yield of PAPs and the solid–liquid ratio. The extraction yield of polysaccharides increased when the solid–liquid ratio increased from 1:10 to 1:15. The high viscosity leads to poor penetration and thus incompletely extracted for PAPs, resulting in low polysaccharide extraction yield (2, 25). With the increase in liquid volume, the solvent viscosity decreased, and the polysaccharide molecules dissolved in the water increased, so the PAPs' extraction yield was significantly improved (26). Nevertheless, the yield decreased with the ratio increasing from 1:15 to 1:30, possibly because with the increase of solvent, the interaction between water and plant internal material molecules may have weakened, resulting in a decrease in polysaccharide yield (27). Therefore, 1:15 was selected as the optimal solid–liquid ratio because the highest yield of 19.85 ± 0.02% was obtained at this ratio.

### 3.2. Response surface data analysis

#### 3.2.1. Statistical analysis of data and model fitting degree

Three factors (enzyme ratio [X_1_], extraction pH value [X_2_], and extraction temperature [X_3_]) were screened and used as individual variables. The ALPs' extraction yield of PAPs (Y) was used as the response value. In this section, a response surface model was established to optimize the extraction parameters of PAPs. According to the principle of Box–Behnken design central combination test, 17 groups of experiments were designed. The central points of the three factors, ratio of cellulase to pectinase, pH, and temperature, were set to be 1:2, 8.0, and 60°C, respectively. The experimental data are listed in Table 2. A second-order polynomial equation of the regression fitting model between Y and X_1_, X_2_, and X_3_ was obtained as follows (5):


(5) $$\begin{array}{ll} Y= & 19.80+0.18X_{1}+0.50X_{2}-0.27X_{3}-0.24X_{1}X_{2}\\ & -0.02X_{1}X_{3}+0.28X_{2}X_{3}-1.40X_{1}^{2}\\ & -1.66X_{2}^{2}-3.11X_{3}^{2} \end{array}$$

Statistical results from Table 3 suggest that the *P*-value of the regression model in this experiment was < 0.0001, and the lack of fit item was larger than 0.05 (*P* = 0.0828 > 0.05), indicating that the model selection had a significant effect on the extraction rate of PAPs, and was suitable for the optimization of PAPs' extraction conditions. In addition, the adjustment determination coefficient (R^2^) of the model was 0.9953, indicating that 99.53% of the results can be explained by the model (28). The calibration determination coefficient (R${}_{\text{adj}}^{2}$) was 0.9892, demonstrating that this model had a small experimental error and there was a high degree of fitting between model predictions and experimental data (29). This experimental model can be used to analyze and predict the enzyme-assisted extraction of PAPs.

**Table 2:** Box–Benkhen design of the independent variables and experimental values of *Potentilla anserina L*. polysaccharides yield (Y).

**No**.	**Factor**			**Y (PAPs extraction yield)/%**
	**X**_1_ **-enzyme ratio**	**X**_2_ **-extraction pH**	**X**_3_**-extraction temperature /**°**C**	
1	−1	−1	0	16.02 ± 0.890
2	1	−1	0	16.78 ± 0.335
3	−1	1	0	17.17 ± 0.470
4	1	1	0	16.98 ± 0.530
5	−1	0	−1	15.38 ± 0.287
6	1	0	−1	15.86 ± 0.398
7	−1	0	1	14.74 ± 0.688
8	1	0	1	15.14 ± 0.736
9	0	−1	−1	14.85 ± 0.430
10	0	1	−1	15.62 ± 0.099
11	0	−1	1	13.88 ± 0.131
12	0	1	1	15.76 ± 0.470
13	0	0	0	19.83 ± 0.519
14	0	0	0	19.57 ± 0.489
15	0	0	0	19.79 ± 0.037
16	0	0	0	19.91 ± 0.910
17	0	0	0	19.88 ± 0.531

**Table 3:** Results of variance analysis of quadratic multinomial simulation.

**Source**	**Sum of square **	**DF**	**Mean square**	**F value**	***P* value**	**Significance**
Model	69.96	9	7.77	164.54	< 0.0001	^**^
X_1_	0.26	1	0.26	5.56	0.0504	
X_2_	2.00	1	2.00	42.34	0.0003	^**^
X_3_	0.60	1	0.60	12.69	0.0092	^**^
X_1_ X_2_	0.23	1	0.23	4.78	0.0651	
X_1_ X_3_	1.60 × 10^−3^	1	1.60 × 10^−3^	0.034	0.8592	
X_2_ X_3_	0.31	1	0.31	6.52	0.0379	^*^
X${}_{1}^{2}$	8.29	1	8.29	175.44	< 0.0001	^**^
X${}_{2}^{2}$	11.54	1	11.54	244.27	< 0.0001	^**^
X${}_{3}^{2}$	40.80	1	40.80	863.70	< 0.0001	^**^
Lack of Fit	0.26	3	0.086	4.76	0.0828	
Pure Error	0.072	4	0.018			
Cor total	70.292	16	R^2^ = 0.9953, R${}_{\text{Adj}}^{2}$ = 0.9892

^*^was significant at the 5% level (*P* < 0.05); ^**^was significant at the 1% level (*P* < 0.01).

Table 3 shows the results of significance tests for the three factors. As shown, the order of the factors influencing the yield based on significance was pH (X_2_) > temperature (X_3_) > ratio of the enzymes (X_1_). The first term X_2_ (*P* = 0.0003), X_3_ (*P* = 0.0092), and the second term X${}_{1}^{2}$ (*P* < 0.0001), X${}_{2}^{2}$ (*P* < 0.0001), and X${}_{3}^{2}$ (*P* < 0.0001) were all lower than 0.01, showing an extremely significant at a level of 1%, indicating that X_2_ and X_3_ had an extremely significant effect on the extraction yield. There was an obvious quadratic relationship between X_1_, X_2_, X_3_, and Y. The effect of X_1_ (*P* = 0.0504 > 0.05) was weak and not significant at the 5% level. The interaction term X_2_X_3_ (*P* = 0.0379) was significant at the 5% level. There was a significant interaction between X_2_ and X_3_. However, the other interaction terms had larger P-values (P_X1X2_ = 0.0651, P_X1X3_ = 0.8592), i.e., the interactions between X_1_ and X_2_, and X_1_ and X_3_ were weak.

#### 3.2.2. Contour plot and response surface plot analyses

**Figure 2:**
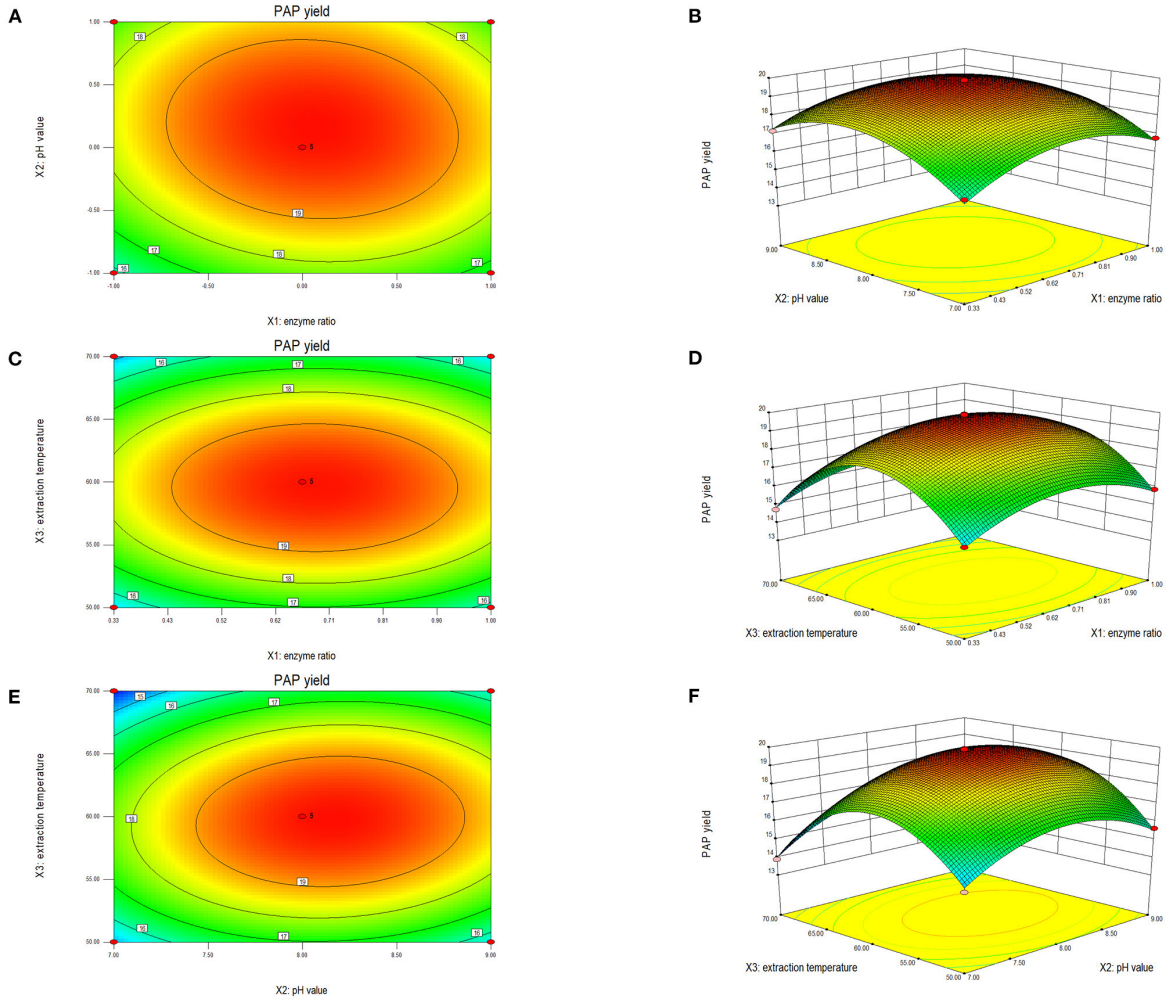
Two-dimensional contour plot and three-dimensional response surface of the ratio of cellulase to pectinase and extraction pH **(A, B)**, enzyme ratio and extraction temperature **(C, D)**, enzyme ratio and extraction temperature **(E, F)** on the extraction yield of *Potentilla anserina L*. polysaccharides.

The shape of a two-dimensional contour graph or that of a three-dimensional response surface graph indicates the interaction strength of the three factors (Figure 2). The interaction strength between any two factors was analyzed systematically to determine the optimal factor level, and thereafter evaluate the effect on the extraction yield of PAPs. Reportedly, the closer a contour line to an ellipse is, the stronger the interaction is, while the closer a contour to a circle is, the weaker the interaction is (18, 30). The steeper the response surface is, the more significant the interaction is. As shown in Figure 2, the response surface of temperature and pH was steep, and the contour line was an ellipse, indicating that both factors had a strong interaction. The other two groups were not significant.

Figures 2A, B shows the interaction effect between the ratio of cellulase to pectinase and pH. The ellipticity shown in Figure 2A was small, indicating a weak interaction between enzyme ratio and pH. The bending degree of the response surface is shown in Figure 2B. The bending degree of pH was steep, and the enzymes' ratio was gentle, indicating that the extraction pH had a significant influence on the response value relative to the enzyme ratio. When the enzymes' ratio was 1:2 and pH was 8.0, the extraction yield of PAPs reached 19.79%.

Figures 2C, D shows the interaction between the ratio of cellulase to pectinase and temperature. According to the contour line shown in Figure 2C, the ellipticity was small, meaning a weak interaction between these two factors. The temperature had a more significant impact on the response value in Figure 2D. When the ratio of the enzymes was 1:2 and the temperature was 60°C, the extraction yield reached up to 19.83%.

As shown in Figure 2E, the contour lines of pH and temperature were elliptical, demonstrating a strong interaction between pH and extraction temperature. The camber of the response surface for temperature and pH value was large in Figure 2F, consistent with the trend shown in Figure 2E. When the temperature was 60°C and the extraction pH was 8.0, the extraction yield of PAPs was 19.83%.

### 3.3. Predictive model and physical model

Through the above analysis conducted using the Design Expert software, the optimal processing conditions of the enzyme-assisted method were obtained as follows: solid–liquid ratio, 1:15; ratio of cellulase to pectinase, 1:2; pH, 8.0; and temperature, 60°C. Under the optimized conditions, the extraction yield of PAPs was 19.80 ± 0.01%. The predicted polysaccharide yield of PAP was 19.84%. The difference between the predicted value and the actual one was little, indicating that this model was suitable for predicting the extraction yield of PAPs. In the previous studies, Shen et al. reported that they extracted the PAPs using the hot water method, and the maximum extraction yield was found at 5.18% (11). Yue et al. extracted PAPs and obtained a PAPs' yield of 9.18% by ultrasound-assisted extraction (31), and Guo et al. extracted PAPs by ultrasound-assisted extraction, and obtained a PAPs' yield of 14.22% (2). Comparing the results of the present study with data published reports, it can be observed that the enzyme-assisted method is more effective in extracting PAPs. However, in the process of extracting, we should take into account the possibility of enzyme activity reducing due to excessive enzyme placement or improper storage; therefore, we should measure enzyme activity before use to ensure the accuracy of the experiment, and appropriately increase the amount of enzyme according to the experimental requirements.

### 3.4. FT-IR analysis

**Figure 3:**
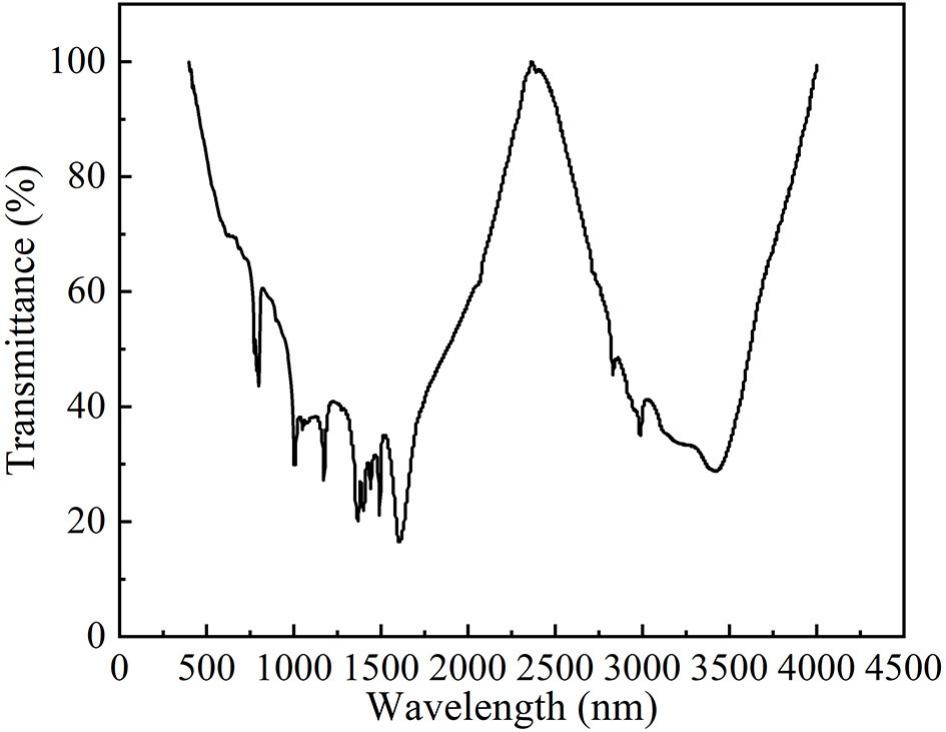
Fourier transform infrared spectroscopy of *Potentilla anserina L*. polysaccharides.

The Fourier transform infrared spectrum of PAPs is shown in Figure 3. The frequency band of approximately 3,422.3 cm^−1^ represents the O-H stretching vibration peak, and the frequency band of approximately 1,375.3 cm^−1^ represents the O-H bending vibration peak. The stretching vibration peak at 2,990.7 cm^−1^ and the bending vibration peak at 1,193.7 cm^−1^ indicated the existence of the O-H bond. The absorption peak at 1,623.5 cm^−1^ was the characteristic peak of N-H bond flexural vibration, indicating the presence of amino sugars in PAPs. The characteristic absorption peak of C=O in the pyran ring was observed at 1,499.4 cm^−1^. The characteristic absorption peak of C-O-C vibration was observed at 798.3 cm^−1^. The results indicated that the PAPs were typical polysaccharides.

### 3.5. The results of SEM

**Figure 4:**
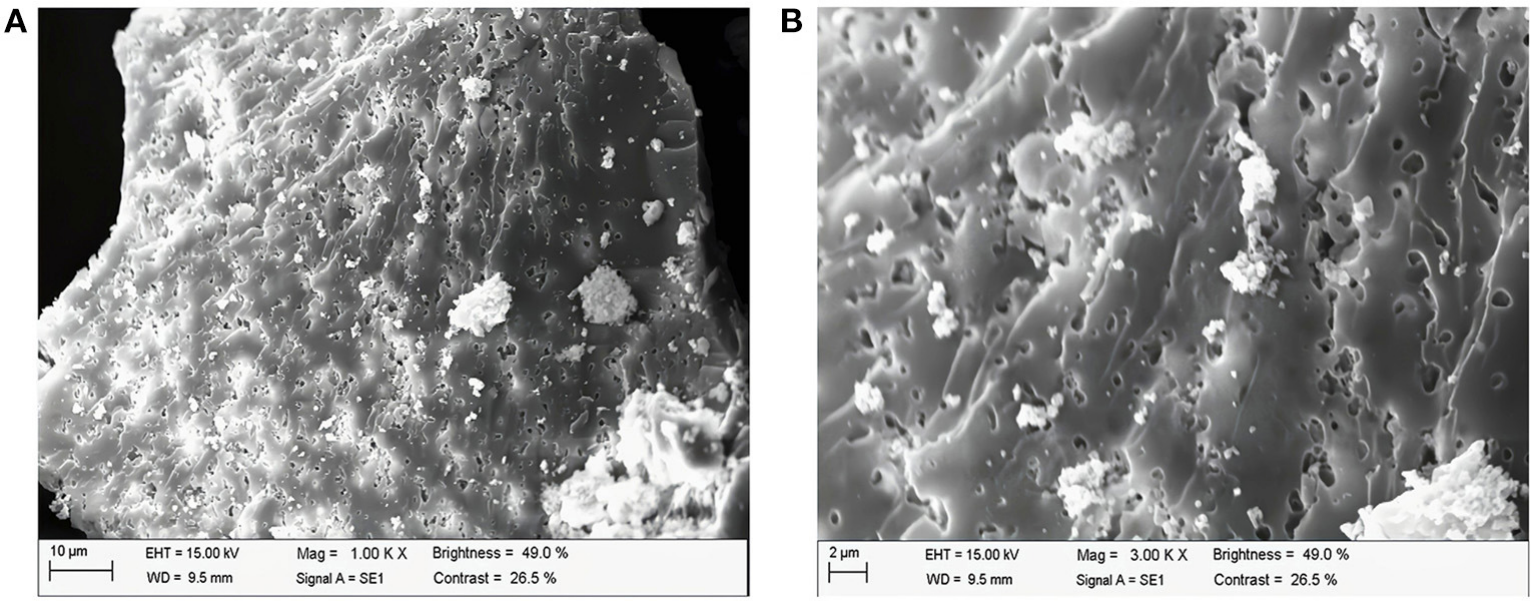
Scanning electron microscopy of *Potentilla anserina L*. polysaccharides **(A)** 1,000 X, **(B)** 3000 X.

Figure 4 shows the images of PAPs at 1,000 × and 3,000 × magnification under a scanning electron microscope. The surface of the PAPs' sample was an irregularly shaped mass with a rough surface and many pores, and attached particles with irregular shape and size (1,000 × , Figure 4A; and 3,000 × , Figure 4B). The results showed that there were large repulsive forces between the molecules of PAPs, resulting in a large number of intermolecular pores. Shi et al. also observed through SEM that the structure of PAP obtained by the water extraction method is granular (32).

### 3.6. The results of HPLC of PAPs

**Figure 5:**
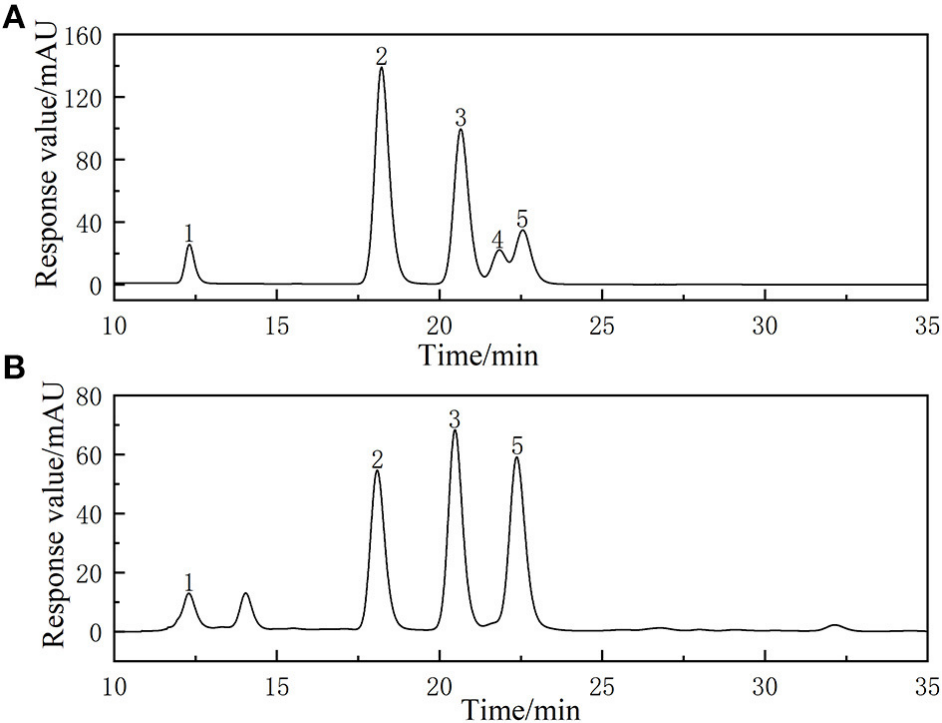
High-performance liquid chromatogram of monosaccharides of reference substances solution **(A)** and monosaccharides composition of *Potentilla anserina L*. polysaccharides **(B)**.

Compared with the chromatogram of the standard solution (Figure 5), the PAPs were found to be a heteropolysaccharide. PAPs were mainly composed of galactose, rhamnose, arabinose, and glucose, alongside a molar ratio of 3.19: 1: 3.06: 2.89. Reportedly, the PAPs extracted by ultrasonic-assisted enzymolysis method mainly consist of galactose, rhamnose, arabinose, and glucose, with a molar ratio of 3.49: 1: 1.37: 2.94 (33). The monosaccharides of the two PAPs were consistent in composition, and the proportion of each component was different. We hypothesized that it might be the different processing methods that caused the differences in polysaccharides (34). Moreover, previous studies have put forward that a treatment method may affect the structure and bioactivity of polysaccharides. The function and activity mechanism of a polysaccharide was closely related to its composition and structure (35).

### 3.7. Antioxidant activity analysis

#### 3.7.1. Hydroxyl radical scavenging

**Figure 6:**
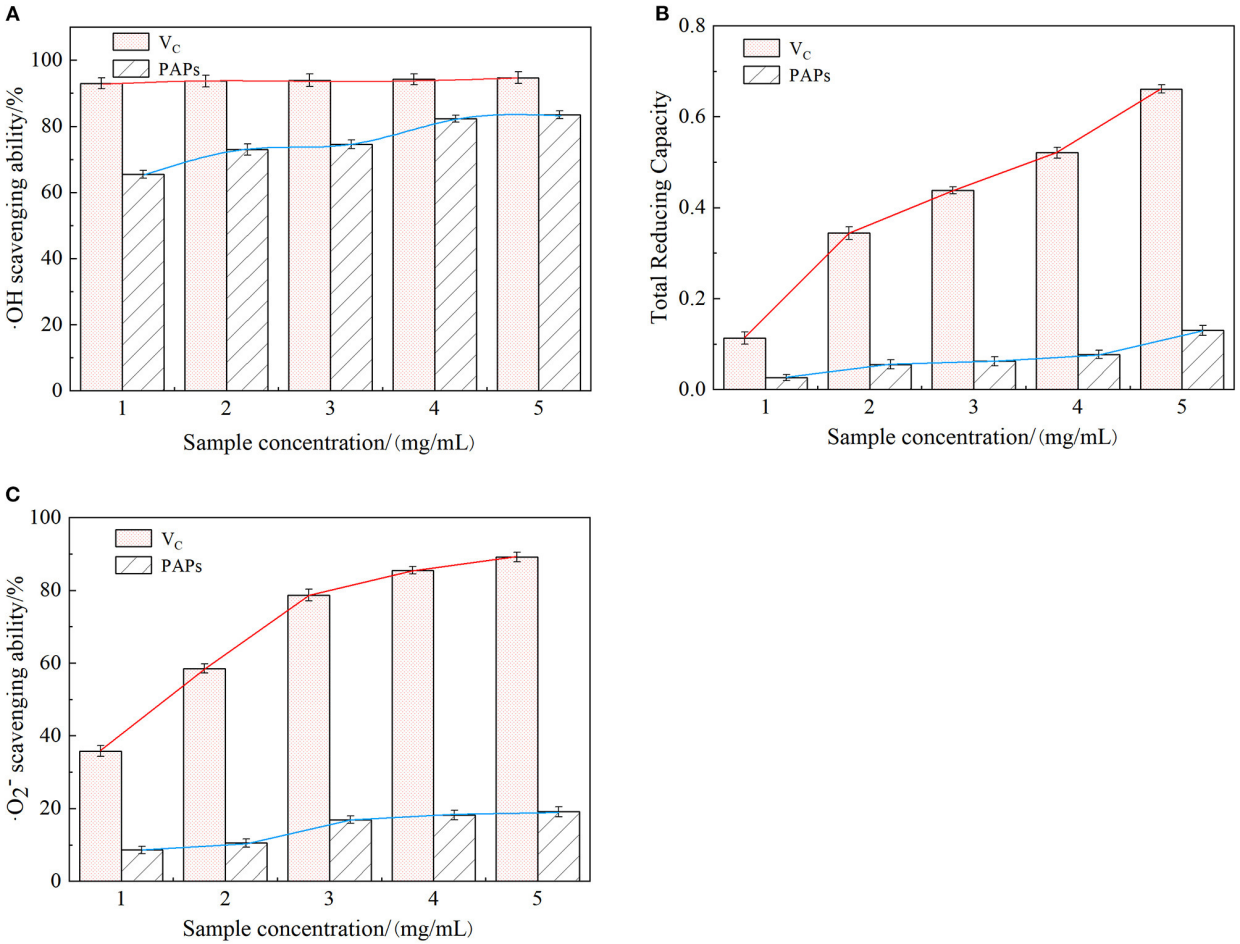
Scavenging effect of *Potentilla anserina L*. polysaccharides on ·OH radicals **(A)**, reducing power **(B)** and ·O${}_{2}^{-}$ radicals **(C)**.

Figure 6A shows the relationship between PAPs' concentration and the scavenging capacity of hydroxyl radicals. The scavenging rate increased over the range of tested concentrations of PAPs. In detail, the hydroxyl radical scavenging capacity rapidly increased with increasing PAPs' concentration from 0 to 0.2 mg/mL, then slowly increased after PAPs surpassed 0.2 mg/mL. A measure of 1.0 mg/mL of PAPs exhibited a maximal scavenging rate of 83.5% with an IC_50_ value of 0.37 mg/mL, close to that of V_C_. Hydroxyl radical (·OH) is one of the free radicals produced by the human body due to continuous contact with the outside world, such as respiration, external pollution, and radiation. It is also one of the most powerful reactive oxygen species (ROS). The ·OH can penetrate biofilms and react with different cell components, causing oxidative damage, cancer, aging, inflammation, and other diseases (36). Therefore, hydroxyl radical scavenging ability is often used as one of the indicators to test antioxidant activity *in vitro* (37). From Figure 6A, the ·OH radical scavenging ability of PAPs was lower than V_C_, but the difference was small. The results indicated that PAPs had a strong scavenging ability of ·OH radical.

#### 3.7.2. Reducing power scavenging

As an electron donor, polysaccharides can react with free radicals to generate stable products that convert Fe^3+^ to Fe^2+^ in the ferricyanide complex, preventing peroxides generation. The stronger the reducing power of polysaccharides is, the higher the corresponding absorbance value is. Reducing power reflected the electron-giving capacity and was also an important indicator of antioxidant activity *in vitro* (38). As shown in Figure 6B, the absorbance values of V_C_ and PAPs were proportional to their concentrations, but the absorbance of PAPs increased slowly, with a smaller increment and a maximum value of only 0.127. These results indicated that PAPs had a certain degree of reducing ability.

#### 3.7.3. Superoxide anion radicals scavenging

As shown in Figure 6C, ·O${}_{2}^{-}$ scavenging capacity of PAPs was increased across the tested concentrations (0–1.0 mg/mL) and varied in a small range (0–19.10%). Superoxide anion radical is a highly toxic reactive oxygen species, playing an important role in the formation of oxygen species (ROS), such as hydrogen peroxide and hydroxyl radicals. Superoxide anion radicals can cause tissue damage and various diseases (39). The IC_50_ value of PAPs was 45.017 mg/mL, and that of V_C_ was 0.295 mg/mL. The results of ·O${}_{2}^{-}$ radical indicated that PAPs had a certain degree of scavenging ability of superoxide anions.

As discussed above, the concentration of PAPs was proportional to the scavenging ability of ·OH radical, reducing power, and ·O${}_{2}^{-}$ radical, demonstrating that the PAPs have the potential as a natural antioxidant.

## 4. Conclusion

The PAPs were efficiently extracted using an enzyme-assisted method with an extraction yield of 19.80 ± 0.01%. The PAPs were α-pyran polysaccharides and were mainly composed of galactose, rhamnose, arabinose, and glucose, with a molar ratio of 3.19: 1: 3.06: 2.89. The PAPs possess strong antioxidant activity, high scavenging ability on hydroxyl free radicals, a certain degree of scavenging ability on superoxide ions, and a satisfactory total reducing ability. To conclude, enzyme-assisted extraction is an efficient method to extract PAPs, and PAPs have the potential as natural antioxidants.

## Data availability statement

The raw data supporting the conclusions of this article will be made available by the authors, without undue reservation.

## Author contributions

PG, JL, and DG designed the experiments for this manuscript. HongC, JM, and YZ prepared PAPs and performed the experimental design of the antioxidant. HongfC and TW performed PAP structural determination. PG and HongC wrote the manuscript. All authors read and approved the final manuscript.
